# The Role of Extracorporeal Shock Wave Therapy in Keloids and Hypertrophic Scars: A Systematic Review

**DOI:** 10.7759/cureus.71869

**Published:** 2024-10-19

**Authors:** Natalia Gili, Kirill Micallef Stafrace, Francesco Laybats, Tiziana Mifsud

**Affiliations:** 1 Surgery, Mater Dei Hospital, Msida, MLT; 2 Sports Medicine, Triagon Academy, Marsa, MLT; 3 Trauma and Orthopedics, Mater Dei Hospital Malta, Msida, MLT; 4 Health Sciences, University of Malta, Msida, MLT

**Keywords:** extracorporeal shock wave therapy, hypertrophic scars, keloids, pathological scars, scars

## Abstract

Keloids and hypertrophic scars are pathological scars that result from a prolonged and aberrant response to wound healing, resulting in raised and thickened skin lesions. Traditional treatments include occlusive dressings, compression therapy, intralesional steroids, and surgical excision with refashioning. Extracorporeal shock wave therapy (ESWT) is a non-invasive treatment method that employs mechanotransduction to stimulate a biological cascade resulting in tissue regeneration. This review aims to explore the literature for published evidence on the role of ESWT in the treatment of keloids and hypertrophic scars.

A literature search following the PRISMA guidelines was conducted on PUBMED, Google Scholar, and the Cochrane Database of Systematic Reviews, for studies demonstrating the use of ESWT in keloids and hypertrophic scars. From this, 79 articles were identified, 12 of which met the eligibility criteria and were thus analyzed and included in the study.

As monotherapy for keloids, ESWT showed comparable improvements in functional and aesthetic outcomes compared to traditional intralesional steroid therapy. Keloids treated with combination therapy of ESWT with intralesional steroids had better outcomes than those treated with intralesional steroids alone. Improvements in hypertrophic scar cosmesis, discomfort, and function with the use of ESWT were reported. Histologic improvements such as decreased collagen content, reduction of fibrotic markers, and fibrogenic cytokines were also described.

ESWT is a promising treatment modality for pathological scars, offering comparable outcomes to traditional therapies with added benefits such as non-invasiveness. Further research is warranted to establish optimal protocols and its role in pathological scar management.

## Introduction and background

Wound healing is a physiological cascade, which occurs in reaction to tissue injury [1]. It can be divided into four phases, namely the hemostatic phase, inflammatory phase, proliferative phase, and maturation or remodeling phase [2]. Scar formation is the end result of wound healing. This normally results in a normotrophic scar, which is flat, pale, and has normal or reduced sensitivity and elasticity [3].

Keloid and hypertrophic scars are pathological scars that result from a prolonged and aberrant response to wound healing, resulting in excessive tissue [4]. They most commonly arise as a result of tissue trauma, which may be intentional as in the case of surgery and body piercings, or due to accidental trauma such as in burns and insect bites. Rarely, they may occur spontaneously. Risk factors include high wound tension, certain anatomical sites, e.g., across joint lines, upper torso, and earlobes, ethnicities with darker skin tones, and ages between 10 and 30 years [5].

Clinically, this results in a raised and thickened scar, which may be pruritic and painful. They may be clinically distinguished by the scar boundaries, history of progression, and association with scar contracture. Keloid scars extend beyond the original wound margins, may develop from three months to several years after tissue injury, and do not exhibit a pattern of regression. Hypertrophic scars are confined to the boundaries of the original wound edges. They arise acutely after the original injury, usually within one to two months, and continue developing over six to eight months after tissue injury, after which they may exhibit spontaneous regression. Hypertrophic scars are associated with scar contractures, whereas keloids do not exhibit this characteristic [6].

The exact pathophysiology of keloids and hypertrophic scars is unknown; however, multiple factors have been discussed in the literature. In normotrophic scars, wounds soften and flatten during the maturation phase due to the simultaneous synthesis and degradation of collagen [7]. In keloids and hypertrophic scars, there is an overproduction of collagen compared to normal skin due to a stronger proliferating activity of pathologic fibroblasts, a higher rate of fibronectin biosynthesis, and a lower rate of apoptosis in pathologic fibroblasts compared to normal skin [5]. Fibrotic markers such as alpha-smooth muscle actin (α-SMA) are elevated in both hypertrophic scars and keloids. An overexpression of growth factor receptors has also been shown to play a role in pathological scar formation, in particular transforming growth factor beta 1 (TGF-β1) type I and II receptors. TGF-β1 is a fibrogenic cytokine, which along with its receptors, results in a potent stimulatory effect on ECM protein synthesis and fibroblast proliferation [8]. In keloid and hypertrophic scars, there is an overexpression of TGF-β1 and α-SMA compared to normal tissue [9]. Stem cells in keloids promote excessive collagen production, abnormal wound healing, and sustain the fibroproliferative activity within keloids, contributing to their pathological growth [10].

Normal skin consists of a dermal layer with collagen, which is parallel to the epidermis. In hypertrophic scars, one finds primarily well-organized type III collagen (collagen type I < type III) oriented parallel to the epidermis, with abundant nodules containing myofibroblasts [11,12]. Keloids are mainly composed of large, poorly organized collagen bundles with no nodules or excess myofibroblasts, with an increased collagen type I (collagen type I > type III) [13].

Histologically, both keloids and hypertrophic scars have a normal epidermis, with an abnormal dermis, which is highly vascularized, infiltrated with inflammatory cells, and with high collagen levels [2,14,15]. Collagen is up to 20 times greater in keloids and up to three times greater in hypertrophic scars compared to normotrophic scars [16].

The current traditional treatment methods for hypertrophic scars and keloids include occlusive dressings, compression therapy, steroids, and surgical excision with refashioning [2]. For keloids, intralesional steroid injections consisting of triamcinolone acetonide are recommended as first-line nonsurgical treatment [17].

Extracorporeal shock wave therapy (ESWT) is a non-invasive mechanical therapeutic method that uses a probe to transmit a mechanical force using high amplitude acoustic shock waves that rapidly cycle between negative and positive pressures onto the area being treated [18]. ESWT may use two types of generators, which are focused and radial. For use in soft tissues, radial ESWT is preferred compared to focused ESWT, this covers a more superficial and a greater surface area, allowing the operator to deliver a lower number of pulses and a shorter treatment session, making the treatment more acceptable to the patient [19]. Although the mechanism of ESWT has not been fully established, the literature suggests that it stimulates healing through mechanotransduction to stimulate a biological cascade, resulting in angiogenesis, increased tissue perfusion, and tissue regeneration [20]. The initial clinical application of ESWT was in the treatment of urolithiasis [21]. Since then, the use of ESWT for the treatment of orthopedic pathologies such as tendinopathies and fasciitis, acute and chronic wounds, ulcers, and burns has also been documented [19,22-25].

## Review

Methods

This literature review was conducted systematically according to the PRISMA guidelines [26]. A literature search was performed across three databases using PUBMED, Google Scholar, and the Cochrane Database of Systematic Reviews. A search was carried out on 18th May using the following terms: (Shockwave[title/abstract] OR "Shock wave"[title/abstract] OR "Shock waves"[title/abstract] OR ESWT[title/abstract] OR "Extracorporeal Shock Wave Therapy"[title/abstract]) AND (Keloid[title/abstract] OR Keloids[title/abstract] OR Hypertrophic[title/abstract] OR "Pathological Scars"[title/abstract])

This literature review aims to research the role of ESWT in keloid and hypertrophic scar treatment, collectively referred to as pathological scars. Our research concerns both types of scars, rather than one single type, due to the overlapping pathophysiology and traditional treatment methods. By identifying literature on both types of scars, the authors aim to potentially better inform the understanding of the use of ESWT in pathological scars.

**Figure 1:**
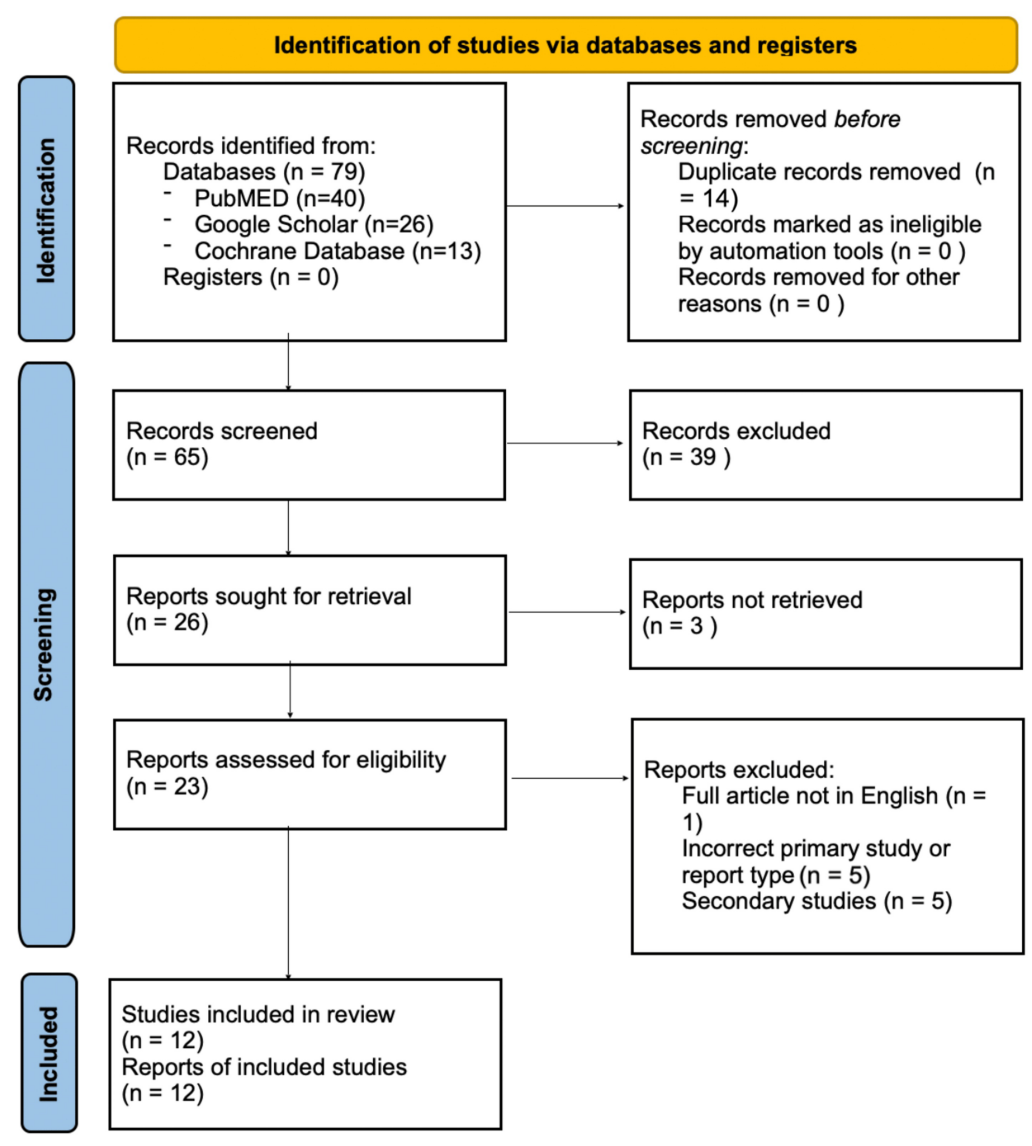
PRISMA flowchart demonstrating the study selection process

A total of 79 research articles were initially identified. The title, abstract, and keywords were independently screened by two authors (NG and KMS) for relevance. Prospective primary studies of any design using ESWT in the context of keloid and hypertrophic scar treatment were included. Duplicate searches, non-English articles, and secondary studies were excluded. No criteria were in place concerning the year of publication. The full-text articles of the potentially relevant studies were retrieved and assessed for eligibility independently by NG and KMS. Discrepancies between reviewers were resolved through discussion. Grey literature was not included in this systematic review due to the difficulty in appraising such non-peer-reviewed sources. The study selection process is outlined in Figure 1.

Results

The results from the 12 included articles were divided into two categories, with two studies concerning keloid scars and 10 studies concerning hypertrophic scars, including seven clinical studies and three preclinical studies, as listed in Table 1.

**Table 1:** List of articles included in the review RCT: randomized controlled trial

List of articles included in the review		
Reference	Study type	Type of pathological scar studied
Wang et al. (2018) [27]	RCT	Keloid scars
Kim et al. (2020) [28]	RCT	Keloid scars
Chuangsuwanich et al. (2022) [29]	RCT	Hypertrophic scars
Fioramonti et al. (2012) [30]	Prospective observational study	Hypertrophic scars
Moorgart et al. (2020) [31]	RCT	Hypertrophic scars
Zaghloul et al. (2016) [32]	RCT	Hypertrophic scars
Lee et al. (2021) [33]	RCT	Hypertrophic scars
Joo et al. (2020) [34]	RCT	Hypertrophic scars
Joo et al. (2017) [35]	RCT	Hypertrophic scars
Zhao et al. (2018) [36]	Animal experimental study	Hypertrophic scars
Cui et al. (2018) [37]	Laboratory-based experimental study	Dermal fibroblasts from hypertrophic scars
Zhao et al. (2018) [38]	Animal experimental Study	Hypertrophic scars

Risk of Bias Assessment

**Figure 2:**
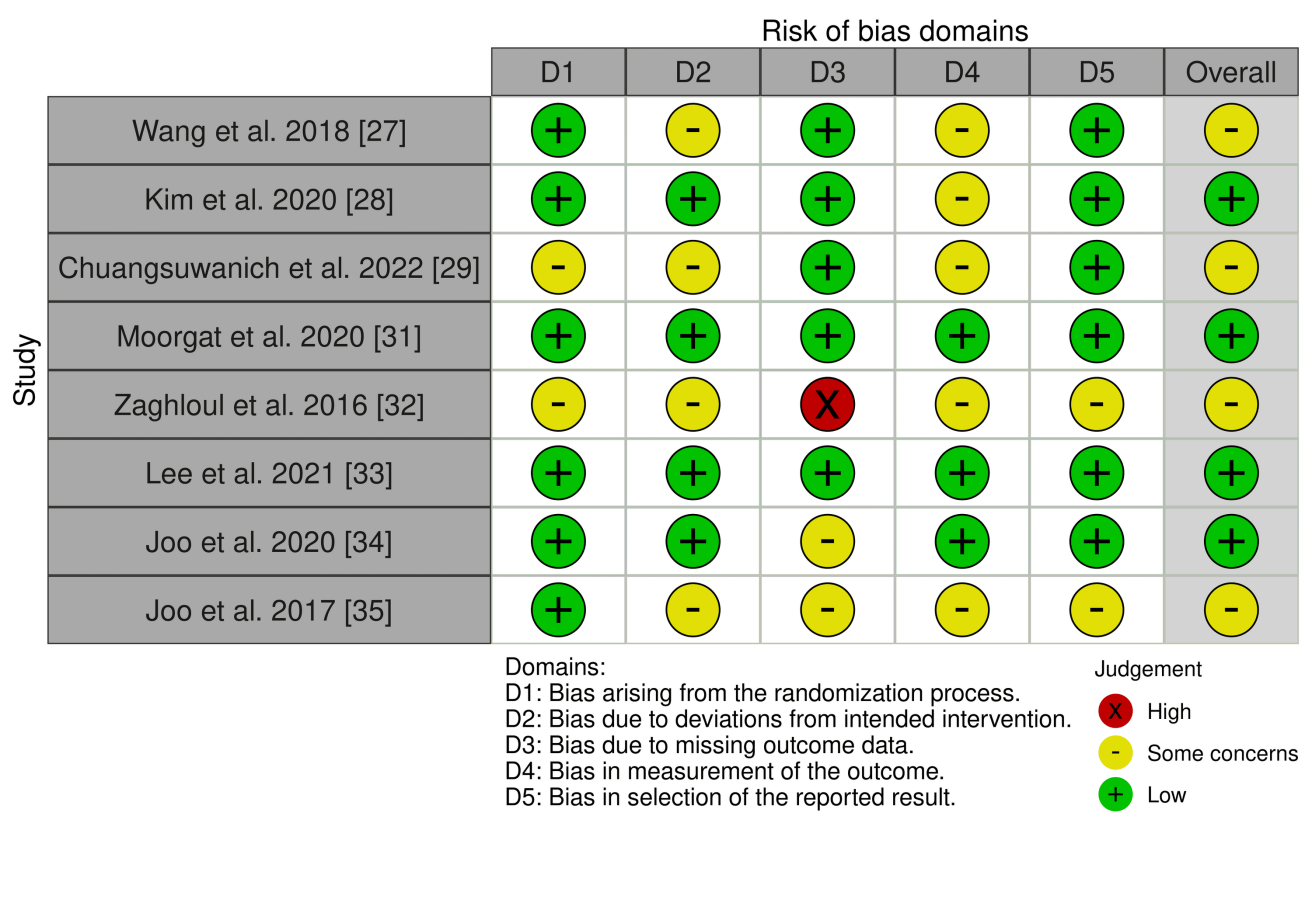
The Cochrane RoB-2 assessment for RCT RoB-2: risk of bias 2; RCT: randomized controlled trial

**Figure 3:**
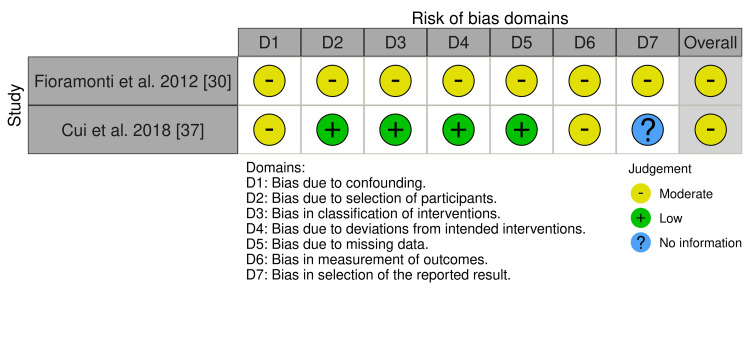
The ROBINS-I RoB assessment for non-RCT ROBINS-I: Risk of Bias in Non-randomized Studies of Interventions; RCT: randomized controlled trial; RoB: risk of bias

**Figure 4:**
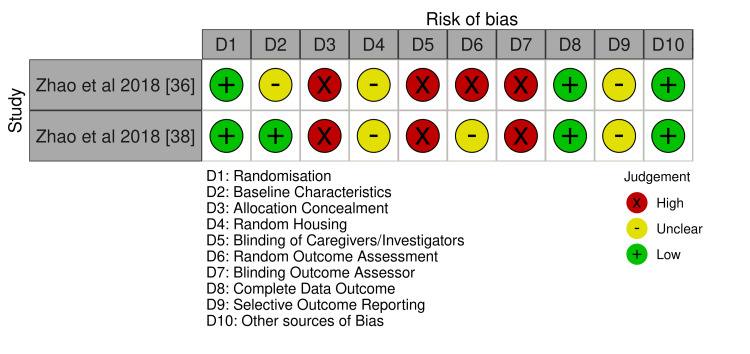
The SYRCLE RoB assessment for animal studies SYRCLE: Systematic Review Centre for Laboratory Animal Experimentation; RoB: risk of bias

A risk of bias (RoB) assessment was done on the 12 articles included in the review. For RCTs, the Cochrane RoB-2 tool was used, as shown in Figure 2. Non-randomized studies were evaluated using the Risk of Bias in Non-randomized Studies of Interventions (ROBINS-I) tool, as depicted in Figure 3. The RoB for the included animal studies was analyzed with the Systematic Review Centre for Laboratory Animal Experimentation (SYRCLE) tool, as presented in Figure 4. To visualize the results, the Robvis tool was used to develop traffic light plots, which are depicted in Figures 2, 3, 4 [39].

Keloids

Two RCTs evaluating the use of ESWT for keloids were identified, as outlined in Table 2. The ESWT treatment characteristics used in studies on keloids are presented in Table 3.

**Table 2:** Study characteristics of studies investigating keloid scars RCT: randomized controlled trial; ESWT: extracorporeal shockwave therapy; SI: steroid injection; VSS: Vancouver scar scale

Keloids - study characteristics			
Reference	Study type	Number of subjects	Outcome measures
Wang et al. (2018) [27]	RCT	39 (22 ESWT, 17 SI)	Gross morphology; functional outcome; local blood flow perfusion; biopsy for histopathological examination and immunohistochemical analysis
Kim et al. (2020) [28]	RCT	40 (20 ESWT+SI, 20 SI only)	VSS; lesion length, width, and height

**Table 3:** ESWT treatment characteristics of studies investigating keloid scars ESWT: extracorporeal shockwave therapy

Keloids - ESWT treatment characteristics						
Reference	Length of treatment	Number of pulses	Density of energy	Frequency	Focused or radial	Instrument used
Wang et al. (2018) [27]	3 sessions over 6 weeks	Lesion size dependent (numbers of impulses = treatment area in cm^2^x8, with a minimum of 500 impulses)	0.11 mJ/mm^2^	4 shocks per second (equivalent to 4Hz)	Not specified	dermPACE
Kim et al. (2020) [28]	1 session per week for 10 weeks	500 impulses/cm^2^	0.29 mJ/mm^2^	250 pulses/min (equivalent to 4.2Hz)	Not specified	dermPACE

An RCT by Wang et al. done in 2017 compared the effectiveness of ESWT with intralesional triamcinolone steroid injection (SI) for the treatment of keloid scars in a group of 39 patients, which were randomly divided [27]. The 22 patients in the ESWT group received three EWST sessions at two weekly intervals. ESWT treatment dosage was lesion size dependent, with the numbers of impulses equal to the treatment area in cm^2^x8, with a minimum of 500 impulses at 0.11 mJ/mm^2^ at a rate of four shocks per second. The 17 patients in the steroid group received three sessions at two weekly intervals using 10 mg intralesional triamcinolone injections.

When comparing the keloid lesions before and after treatments, both the ESWT group and the SI group showed a significant improvement in the total surface area and volume of the lesions. There was a statistically significant improvement in pain (EWST, p=0.035; steroids, p=0.022), Patient and Observer Scar Assessment Scale (POSAS) patient scale (EWST, p=<0.01; SI, p=<0.01) and POSAS observer scale (EWSL p=<0.01; SI p=<0.01) (31). However, there was no significant difference between the results of the two groups (pain, p=0.429; POSAS patient scale, p=0.55; POSAS observer scale, p=0.25) indicating a comparable outcome between ESWT and SI treatment.

Both ESWT and SI groups showed no significant difference in blood flow perfusion before and after treatment (EWST, p=0.540; steroids, p=0.237). Histopathological and immunohistological analysis showed comparable cell activity, proliferation, apoptosis and concentration, angiogenesis, proliferative, anti-inflammatory, and apoptosis biomarkers between both groups, with no statistical significance before and after treatment.

On immunohistochemical analysis, there was a statistically significant decrease (p<0.05) of collagen I content and Masson’s trichome only in the ESWT group, and of collagen III in both the ESWT and steroid groups. MMP-13 levels were increased in both the EWST and SI groups, with a more significant increase in the ESWT group (EWSL, p=<0.001; SI, p=<0.05).

An RCT by Kim et al. in 2019 also looked at the use of EWST in keloids; however, unlike the previous study, EWST was not studied as a monotherapy, but rather as a part of combination therapy in conjunction with intralesional triamcinolone SI [28]. Group A consisted of 20 patients treated with both EWST and SI, while group B consisted of 20 patients treated with SI only. EWST treatment consisted of 10 sessions at weekly intervals at a dose of 500 impulses/cm^2^ at 0.29 mJ/mm^2^ of energy and a frequency of 250 impulses/min, while SI treatment consisted of four sessions at three weekly intervals using 10 mg of triamcinolone per 1 cm^2^ of the lesion.

By the end of the treatments, both groups showed a statistically significant decrease (p<0.05) in the lesions’ lengths, widths, and heights; however, the percentage reductions in each parameter were greater in the group treated with both ESWT and SI than in the group treated with SI only. Both groups showed a percentage decline in the Vancouver scar scale (VSS) (ESWT+SI decline of 56.13%±18.12%) compared with group B (decline of 40.15±14.09%), with a more significant decrease in the group treated with ESWT+SI (p=0.003). These two studies indicate that ESWT has a positive effect on the treatment of keloid scars.

Hypertrophic Scars

The study characteristics of the seven studies regarding hypertrophic scars are shown in Table 4. Furthermore, the ESWT treatment characteristics are presented in Table 5.

**Table 4:** Study characteristics of studies investigating hypertrophic scars RCT: randomized controlled trial; ESWT: extracorporeal shockwave therapy; POSAS: Patient and Observer Scar Assessment Scale; VAS: visual analog scale; TEWL: transepidermal water loss; VSS: Vancouver scar scale

Hypertrophic scars - study characteristics			
Reference	Study type	Number of subjects	Outcome measures
Chuangsuwanich et al. (2022) [29]	RCT	29 patients ESWT	POSAS; erythema index; melanin index; scar area; scar thickness
Fioramonti et al. (2012) [30]	Prospective observational study	16 patients ESWT	Appearance on VAS
Moorgart et al. (2020) [31]	RCT	40 patients (20 ESWT; 20 control)	POSAS; scar color; TEWL; vertical elasticity
Zaghloul et al. (2016) [32]	RCT	40 patients (20 ESWT+20 control)	Ultrasonographic measurement of scar thickness; VSS
Lee et al. (2021) [33]	RCT	48 patients (25 ESWT, 28 control)	Skin thickness; melanin erythema; TEWL; sebum levels; skin elasticity levels
Joo et al. (2020) [34]	RCT	48 patients (23 ESWT, 25 control)	VAS; VSS; ultrasonographic measurement of scar thickness; hand function (Jebsen-Taylor hand function test; grip strength; Perdue pegboard test; and the Michigan hand outcomes questionnaire)
Joo et al. (2018) [35]	RCT	46 patients (23 ESWT, 23 Control)	Numerical rating scale; 5D-itch scale Leuven itch scale; laser Doppler blood perfusion imaging

**Table 5:** ESWT treatment characteristics of studies investigating hypertrophic scars ESWT: extracorporeal shockwave therapy

Hypertrophic scars - ESWT treatment characteristics						
Reference	Length of treatment	Number of pulses	Density of energy	Frequency	Focused or radial	Instrument used
Chuangsuwanich et al. (2022) [29]	1 session per week for 6 weeks	350 +/- 10 pulses cm^2^	0.1 mJ/mm^2^	4 Hz	Not specified	Dermagold 100
Fioramonti et al. (2012) [30]	2 sessions per week for 6 weeks	100 pulses	0.037 mJ/mm^2^	4 Hz	Not specified	Evotron
Moorgart et al. (2020) [31]	1 session per week for 10 weeks	30-50 pulses per cm²	0.25 mJ/mm²	6 Hz.	Not specified	Duolith®-SD1 T-Top
Zaghloul et al. (2016) [32]	2 sessions per week for 6 weeks	2500-3000 pulses	Not specified	Not specified	Not specified	Not specified
Lee et al. (2021) [33]	1 session per week for 6 weeks	100 pulses/cm^2^ (1000-2000 pulses per session)	0.05 to 0.30 mJ/mm^2^	4 Hz	Not specified	Duolith SD-1
Joo et al. (2020) [34]	1 session per week for 4 weeks	100 pulses/cm^2^ (1000-2000 pulses per session)	0.05 to 0.30 mJ/mm^2^	4 Hz	Not specified	Duolith SD-1
Joo et al. (2018) [35]	1 session per week for 3 weeks	100 pulses/cm^2^ (1000-2000 pulses per session)	0.05 to 0.20 mJ/mm^2^	4 Hz	Focused	Duolith SD-1

The two main scar evaluation scores used were the POSAS and the VSS. In a 2022 study by Chuangsuwanich et al., there was a statistically significant improvement (p<0.05) in the parameters of the POSAS for both patient and observer scores after ESWT [29]. Similarly, Moorgart et al. reported a statistically significant difference in the POSAS patient scale after three months (P=0.045) and after six months (P=0.013) and overall POSAS observer scale (p=0.027) [31].

Moorgart et al. demonstrated a statistically significant effect (p=0.011) on objectively measured skin elasticity. However, Lee et al. did not find a significant difference in this parameter [31].

A significant decrease in VSS (p=0.0001) was demonstrated by Zaghloul et al. with a mean decrease of 4.25, equal to an improvement of 48.57% [32]. Joo et al. also reported VSS scores but only found a statistically significant improvement in the vascularity component (p=0.0015), not in the total VSS score (p=0.19) [34].

The VAS (visual analog scale) was used to subjectively measure scar improvement. Joo et al. used the VAS to measure self-reported scar pain severity, finding a statistically significant decrease in pain (p=0.001) [34]. Fioramonti et al. used the VAS to measure subjective scar appearance parameters, with some patients reporting improvement; however, no statistical analysis was provided [30].

An improvement in ultrasound-measured scar thickness with ESWT was demonstrated. Zaghloul et al. revealed a mean reduction of 2.86 mm (42.55%) of scar thickness in scars treated with ESWT [32]. When compared to scars treated with a placebo (mean reduction of 0.8 mm; 12%), there was a statistically significant difference between the ESWT and control group (p=0.0001). Joo et al. supported these findings, also demonstrating a statistically significant improvement in ultrasound-measured scar thickness in the ESWT group compared to the control group (p=0.018) [34]. Lee et al. similarly demonstrated a statistically significant reduction in scar thickness with ESWT (p=0.03) [33].

Lee et al. also reported increased sebum levels in scares treated with ESWT (p=0.02) and an improvement in scar erythema, with a statistically significant value of p=0.03 [33]. However, no statistically significant improvement in scar erythema was demonstrated by Chuangsuwanich et al. (p=0.7) or Moorgart et al. [29,31].

No statistically significant change in transepidermal water loss (TEWL) was demonstrated with ESWT use by Moorgart et al. and Lee et al. (p=0.94) [31,33]. No statistically significant change in measured melanin index was shown by Chuangsuwanich et al. (p=0.87) and Lee et al. (p=0.62); however, an improvement in subjective scar color as a part of the POSAS score (p=<0.01) was noted by Chaungsuwanich [29,33].

EWST has been shown to improve scar pruritus. Joo et al. measured itch frequency, duration, severity, and consequences using a numerical rating scale, Leuven itch scale, and 5D pruritus scale, with all factors showing a statistically significant improvement with p-values of <0.05 for all the measured parameters [35].

Joo et al. demonstrated a statistically significant improvement in hand function in individuals with hypertrophic scars treated with ESWT for the subtasks of card shuffling and picking up small objects; however, no significant change was noted for the other subtasks studied [35].

Preclinical Studies

Three preclinical studies were identified and the study and treatment characteristics are presented in Table 6 and Table 7. These studies demonstrated a reduction in markers of fibrotic disease in hypertrophic scars. Since such markers are elevated in both hypertrophic scars and keloids, these studies suggest the potential applicability of these findings to keloid tissue.

**Table 6:** Study characteristics of preclinical studies investigating hypertrophic scars L-ESWT: low-energy extracorporeal shock wave therapy; H-ESWT: high-energy extracorporeal shock wave therapy; TGF-β1: transforming growth factor-beta 1; α-SMA: alpha-smooth muscle actin; Smad: mothers against decapentaplegic homolog

Preclinical studies - study characteristics		
Reference	Number of subjects	Assessment of scar improvement
Zhao et al. (2018) [36]	96 rabbits (32 L-ESWT, 32 H-ESWT, 32 control)	Scar elevation index; fibroblast density; collagen fiber arrangement
Cui et al. (2018) [37]	Dermal fibroblast culture	Expression of TGF-β1, α-SMA, collagen I and III, fibronectin, and protein expression
Zhao et al. (2018) [38]	24 rabbits (8 L-ESWT, 8 H-ESWT, 8 control)	Expression of TGF-β1, Smad2, Smad3 and Smad 7, scar wrinkles, hemoglobin, scar measurements, melanin

**Table 7:** ESWT treatment characteristics of preclinical studies investigating hypertrophic scars ESWT: extracorporeal shockwave therapy; L-ESWT: low-energy extracorporeal shock wave therapy; H-ESWT: high-energy extracorporeal shock wave therapy

Preclinical studies - ESWT treatment characteristics						
Reference	Length of treatment	Number of pulses	Density of energy	Frequency	Focused or radial	Instrument used
Zhao et al. (2018) [36]	1 session per week for 4 weeks	500 pulses	L-ESWT at 0.1 mJ/mm^2^, H-ESWT at 0.2 mJ/mm^2^	8 Hz	Not specified	Swiss DolClast Classic
Cui et al. (2018) [37]	1 session	1000 pulses	0.03, 0.1, 0.3 mJ/mm^2^	4Hz	Focused	Duolith SD-1
Zhao et al. (2018) [38]	1 session per week for 4 weeks	500 pulses	L-ESWT at 0.1 mJ/mm^2^, H-ESWT at 0.18 mJ/mm^2^	8 Hz	Radial	Swiss DolClast Classic

Zhao et al. reported an improvement in collagen arrangement, with collagen fibers being thinner and loosely organized in ESWT-treated tissue [36]. Additionally, there was a significant reduction in scar elevation index (P=0.010 for low-energy extracorporeal shock wave therapy (L-ESWT) and P=0.026 for high-energy extracorporeal shock wave therapy (H-ESWT)) and a reduction in fibroblast density. Immunohistochemistry analysis revealed that ESWT suppressed PCNA, a marker of cell proliferation and reduced α-SMA expression, a marker of myofibroblast formation. Myofibroblasts demonstrate the ability to increase collagen synthesis and contractile activity, therefore, playing a key role in fibrotic disease.

Cui et al. demonstrated a reduction in TGF-β1 mRNA level and protein expression in hypertrophic scar fibroblasts treated with ESWT treatment compared to nontreated cells (p<0.05) [37]. Furthermore, this study demonstrated a significant decrease in α-SMA mRNA expression and vimentin expression (p<0.05). The study also showed a reduction in the expression of extracellular matrix proteins such as collagen-1 and fibronectin in HTSF (p<0.05). N-cadherin expression decreased (p<0.05), while E-cadherin expression increased (p<0.05).

In a study by Zhao et al., improved scar texture was observed, with a decrease in wrinkles and hemoglobin in hypertrophic scar tissue treated with L-ESWT [38]. Additionally, Smad3, a protein involved in the TGF-β1/Smad pathway, was significantly inhibited with L-ESWT treatment (p=<0.05). A reduction in smad3 expression is associated with the reduction of collagen I synthesis and improved wound healing, thus improving hypertrophic scar appearance.

A summary of the studies comparing the ESWT treatment methods and biological, functional, and aesthetic outcomes is demonstrated in Table 8.

**Table 8:** Summary of ESWT treatment methods and biologic, functional, and aesthetic outcome results RCT: randomized controlled trial; ESWT: extracorporeal shockwave therapy; MMP13: matrix metallopeptidase 13; POSAS: patient and observer scar assessment scale; VAS: visual analog scale; TEWL: transepidermal water loss; VSS: Vancouver scar scale; L-ESWT: low-energy extracorporeal shock wave therapy; H-ESWT: high-energy extracorporeal shock wave therapy; TGF-β1: transforming growth factor-beta 1; α-SMA: alpha smooth muscle actin; Smad: mothers against decapentaplegic homolog

Study		ESWT treatment characteristics					Outcome results		
Reference	Study type	Length of treatment	Number of pulses	Density of energy	Frequency	Focused or unfocused	Biological clinical outcome results	Functional clinical outcome results	Aesthetic clinical outcome results
Wang et al. (2018) [27]	RCT	3 sessions over 6 weeks	Lesion size dependent (numbers of impulses=treatment area in cm^2^x8, with a minimum of 500 impulses)	0.11 mJ/mm^2^	4 shocks per second (equivalent to 4 Hz)	Not specified	Statistically significant improvement in collagen I content, Masson’s trichome, collagen III, and MMP-13 levels. No statistically significant improvement in blood flow perfusion, cell activity, proliferation, apoptosis and concentration, angiogenesis, proliferative, anti-inflammatory, and apoptosis biomarkers	Statistically significant improvement in functional outcomes in POSAS patient scale and observer scale	Statistically significant improvement in total surface area and volume of the lesions; aesthetic outcomes of POSAS patient scale and observer scale
Kim et al. (2020) [28]	RCT	1 session per week for 10 weeks	500 impulses/cm^2^	0.29 mJ/mm^2^	250 pulses/min (equivalent to 4.2 Hz)	Not specified	None assessed	None assessed	Statistically significant improvement in lesion length, width, and height, and VSS
Chuangsuwanich et al. (2022) [29]	RCT	1 session per week for 6 weeks	350 +/- 10 pulses/cm^2^	0.1 mJ/mm ^2^	4 Hz	Not specified	None assessed	Statistically significant improvement in functional outcomes in POSAS patient scale and observer scale	Statistically significant improvement in aesthetic outcomes of POSAS patient scale and observer scale. No statistically significant improvement in erythema index, melanin index
Fioramonti et al. (2012) [30]	Prospective observational study	2 sessions per week for 6 weeks	100 pulses	0.037 mJ/mm^2^	4 Hz	Not specified	None assessed	None assessed	Improvement in VAS (no statistical analysis available)
Moorgart et al. (2020) [31]	RCT	1 session per week for 10 weeks	30-50 pulses per cm²	0.25 mJ/mm²	6 Hz	Not specified	Statistically significant improvement in vertical elasticity. No statistically significant improvement in TEWL	Statistically significant improvement in functional outcomes in POSAS patient scale and observer scale	Statistically significant improvement in aesthetic outcomes of POSAS patient scale and observer scale. No statistically significant improvement in color
Zaghloul et al. (2016) [32]	RCT	2 sessions per week for 6 weeks	2500-3000 pulses	Not specified	Not specified	Not specified	None assessed	None assessed	Statistically significant improvement in ultrasound measurement of scar thickness and VSS
Lee et al. (2021) [33]	RCT	1 session per week for 6 weeks	100 pulses/cm^2 ^(1000-2000 pulses per session)	0.05 to 0.30 mJ/mm^2^	4 Hz	Not specified	Statistically significant improvement in sebum levels. No statistically significant improvement in TEWL, skin elasticity	None assessed	Statistically significant improvement in scar thickness, erythema. No statistically significant improvement in the melanin index
Joo et al. (2020) [34]	RCT	1 session per week for 4 weeks	100 pulses/cm^2^ (1000-2000 pulses per session)	0.05 to 0.30 mJ/mm^2^	4 Hz	Not specified	Statistically significant improvement in skin vascularity	Statistically significant improvement in pain, improved hand function	Statistically significant improvement in scar thickness, VAS. No statistically significant improvement in scar pigmentation, total VSS score
Joo et al. (2018) [35]	RCT	1 session per week for 3 weeks	100 pulses/cm^2 ^(1000-2000 pulses per session)	0.05 to 0.20 mJ/mm^2^	4 Hz	Focused	Statistically significant improvement in the numerical rating scale, laser Doppler blood perfusion imaging	Statistically significant improvement in the numerical rating scale, 5D-itch scale, and Leuven Itch scale	None assessed
Zhao et al. (2018) [36]	Preclinical study	1 session per week for 4 weeks	500 pulses	L-ESWT at 0.1 mJ/mm^2^, H-ESWT at 0.2 mJ/mm^2^	8 Hz	Not specified	Statistically significant improvement in collagen fiber arrangement, fibroblast density	None assessed	Statistically significant improvement in scar elevation index
Cui et al. (2018) [37]	Preclinical study	1 session	1000 pulses	0.03, 0.1, 0.3 mJ/mm^2^	4 Hz	Focused	Statistically significant improvement in TGF-β1, α-SMA, collagen I and III, fibronectin, and protein expression	None assessed	None assessed
Zhao et al. (2018) [38]	Preclinical study	1 session per week for 4 weeks	500 pulses	L-ESWT at 0.1 mJ/mm^2^, H-ESWT at 0.18 mJ/mm^2^	8 Hz	Radial	Statistically significant improvement in hemoglobin, TGF-β1, Smad2, Smad3, Smad7	None assessed	Statistically significant improvement in scar wrinkles. No statistically significant improvement in scar diameter, area, elevation of volume, and melanin

Discussion

The findings from the studies on the use of ESWT for keloid and hypertrophic scars suggest important clinical implications and open new potential options for pathological scar management.

The studies on keloid scars suggest that the biological, functional, and aesthetic results of scars treated with ESWT are comparable to those treated with intralesional SIs, the current gold standard treatment for keloids. This suggests that ESWT could serve as an alternative approach for patients who may not tolerate steroids well, have contraindications to steroid treatment, or prefer a non-invasive treatment option. ESWT was shown to potentially act synergistically when used as a combination therapy with SI treatment, resulting in better outcomes than SI treatment alone. The implications here are significant, as ESWT could potentially reduce the need for repeated SIs, which are associated with various side effects, including patient discomfort, skin atrophy, skin hypopigmentation, and systemic absorption issues [40]. ESWT could be integrated into multi-modal treatment regimens, potentially improving overall treatment efficacy and patient satisfaction.

The review of seven studies on hypertrophic scars, which share pathophysiological characteristics with keloids, further supports the utility of ESWT in pathological scars. Across these studies, significant improvements were observed in scar appearance, elasticity, and thickness, as measured by various scales, including POSAS and VSS. This indicates that ESWT could potentially be of benefit in treating the cosmetic appearance of hypertrophic scars.

The improvements in subjective measures like scar pruritus and hand function in patients with hypertrophic scars treated with ESWT also suggest potential benefits in functional improvements. This may have significant implications for patient quality of life, particularly for patients whose scars interfere with daily activities.

Inconsistent results concerning scar erythema and improvements in sebum levels were reported, while no significant change in transepidermal water loss or melanin index was observed. This indicates that ESWT might be more effective in treating certain scar characteristics than others.

The preclinical studies included in the review offer insights into the mechanisms underlying the effects of ESWT on scar tissues, namely the reduction in markers of fibrotic disease and improvements in collagen arrangement and scar texture. This further supports the use of ESWT in pathological scar management, by demonstrating its influence on the underlying cellular processes that drive pathologic scar formation.

These results suggest that ESWT may not be a one-size-fits-all solution, and its effectiveness could depend on specific scar characteristics or patient factors. The variability in outcomes and results across studies highlights the need for further research to optimize treatment protocols, such as energy levels, impulse frequency, and treatment duration. This will help the clinician to improve outcomes by aligning treatment more closely with the unique features of each scar, keeping in mind the role of ESWT in pathological scar management.

Limitations

Only a small number of studies on the use of ESWT for keloid and hypertrophic scars were available. These studies often had small sample sizes, varying outcomes, and methodologies, therefore restricting the extrapolation of the findings. There is a lack of long-term follow-up data and so the long-term effect of ESTW treatment on pathologic scars remains uncertain. Publication bias was not formally assessed in this review due to the relatively small number of studies included.

We suggest that future research should focus on conducting RCTs with standardized treatment protocols and outcome measures, and extended follow-up periods to enable potential assessments of heterogeneity and meta-analyses, and potentially allow identification of optimal treatment protocols. 

## Conclusions

ESWT is a promising treatment modality for hypertrophic scars and keloids, offering comparable outcomes to traditional therapies with added benefits such as non-invasiveness. It has shown potential in improving scar appearance and symptomatology such as pruritus and pain. Further research is warranted to establish optimal protocols for the use of ESWT and its role in pathological scar management.
